# Temozolomide Induces the Acquisition of Invasive Phenotype by O6-Methylguanine-DNA Methyltransferase (MGMT)^+^ Glioblastoma Cells in a Snail-1/Cx43-Dependent Manner

**DOI:** 10.3390/ijms22084150

**Published:** 2021-04-16

**Authors:** Paweł Kochanowski, Jessica Catapano, Maciej Pudełek, Tomasz Wróbel, Zbigniew Madeja, Damian Ryszawy, Jarosław Czyż

**Affiliations:** Department of Cell Biology, Faculty of Biochemistry, Biophysics and Biotechnology, Jagiellonian University in Kraków, ul. Gronostajowa 7, 30-387 Kraków, Poland; pawel.kochanowski@student.uj.edu.pl (P.K.); 1catapanojessica@gmail.com (J.C.); maciej.pudelek@doctoral.uj.edu (M.P.); 1tomaszwrobel@gmail.com (T.W.); z.madeja@uj.edu.pl (Z.M.); damian.ryszawy@uj.edu.pl (D.R.)

**Keywords:** Cx43, glioblastoma multiforme, drug-resistance, MGMT, Snail-1, microevolution

## Abstract

Glioblastoma multiforme (GBM) recurrences after temozolomide (TMZ) treatment result from the expansion of drug-resistant and potentially invasive GBM cells. This process is facilitated by O6-Methylguanine-DNA Methyltransferase (MGMT), which counteracts alkylating TMZ activity. We traced the expansion of invasive cell lineages under persistent chemotherapeutic stress in MGMT^low^ (U87) and MGMT^high^ (T98G) GBM populations to look into the mechanisms of TMZ-induced microevolution of GBM invasiveness. TMZ treatment induced short-term, pro-invasive phenotypic shifts of U87 cells, in the absence of Snail-1 activation. They were illustrated by a transient induction of their motility and followed by the hypertrophy and the signs of senescence in scarce U87 sub-populations that survived long-term TMZ stress. In turn, MGMT^high^ T98G cells reacted to the long-term TMZ treatment with the permanent induction of invasiveness. Ectopic Snail-1 down-regulation attenuated this effect, whereas its up-regulation augmented T98G invasiveness. MGMT^low^ and MGMT^high^ cells both reacted to the long-term TMZ stress with the induction of Cx43 expression. However, only in MGMT^high^ T98G populations, Cx43 was directly involved in the induction of invasiveness, as manifested by the induction of T98G invasiveness after ectopic Cx43 up-regulation and by the opposite effect after Cx43 down-regulation. Collectively, Snail-1/Cx43-dependent signaling participates in the long-term TMZ-induced microevolution of the invasive GBM front. High MGMT activity remains a prerequisite for this process, even though MGMT-related GBM chemoresistance is not necessary for its initiation.

## 1. Introduction

The phenotypic microevolution of tumor cells determines the promotion and progression of tumors, and it is crucial for their lethality. It is manifested by selective survival/expansion of cell lineages differing in sensitivity to extrinsic stress. Multiple signaling pathways that govern this process depend on intercellular communication systems established within the tumor cell populations and between tumor and “stromal” cells [1]. The mediators of this communication fall into three basic categories: (i) “endo/paracrine” or humoral factors; (ii) juxtacrine signals/exchange of mechanical stimuli via membrane receptors [2,3,4]; and, (iii) direct exchange of metabolites/organelles via gap junctions/nanotunnels [5,6,7]. These signaling systems can trigger secular and/or heritable phenotypic switches in “metastable” cells that potentially enhance cell invasiveness and resistance to chemotherapeutic stress. Membrane pumps that reduce the intracellular levels of chemotherapeutics [8], the autophagy of damaged organelles, and multiple DNA repair systems determine drug-resistance [9]. The correlation of their efficiency with the pro-invasive phenotypic switches of tumor cells, such as epithelial-mesenchymal transition (EMT; [10]) leads to the recruitment of drug-resistant cells to the invasive front(s) of tumors [11,12,13]. Even though the acquired drug-resistance results in the ultimate recurrence of malignant tumors after their temporary remissions, and it is a major challenge of current clinical oncology [14], the interrelations between its microevolution and the formation of tumor invasive front(s) remain obscure.

In particular, a rapid development of invasiveness/drug-resistance after therapeutic cycles determines the swift recurrences of GBM [15,16,17,18,19,20]. The median survival time of diagnosed GBM patients ranges between 12 and 14 months, and it remained constant over the recent decades [21,22]. Accordingly, the effective GBM treatment is regarded as the most prominent challenge for contemporary neurooncology. Problems with the treatment result from the brain tissue anatomy, which limits the efficient administration of the drugs [23]. The currently available treatment strategies often prompt the microevolution and selective expansion of drug-resistant and potentially invasive GBM lineages. Consequently, invasive cells can effectively proliferate and spread after the cessation of chemotherapy. In GBM, this process is governed by glial-to-mesenchymal transition (GMT; an analogue of EMT in cancers), and it results in the invasive front formation. EMT is a sequence of events that result in the acquisition of rear-front polarity, high nanomechanical elasticity, and motility by originally immobile epithelioid cells. These events are controlled by a set of transcription factors (incl. Snail-1, Twist, ZEB-1, Slug). They activate the shifts in bioenergetic homeostasis, rearrangements of cytoskeleton architecture, cellular susceptibility to chemotactic signals, and extracellular matrix remodeling [24,25,26]. Mesenchymal markers are expressed in high-grade brain tumors [27] and EMT-related phenotypic changes are often seen at their invasion fronts [28,29]. Among these factors, Snail-1 has been implicated in the regulation of EMT-related processes in brain tumors, incl. glioma progression [30,31,32,33,34,35].

Similarly to Snail-1, connexin(Cx)43 has been pinpointed as important for GBM progression. Cx43 constitutes gap junctional channels, which mediate the intercellular exchange of small (<1 kDa) molecules in the process of gap junctional intercellular coupling (GJIC) [7]. The deficiency of tissue-specific connexins selectively promotes the proliferation of early tumor cells, whereas Cx43 re-expression often accompanies the formation of tumor invasive fronts. Cx43 also regulates the induction of epithelial-mesenchymal transition (EMT) via the activating effect on Snail-1 expression [36,37]. These functions are executed both via channel-dependent and channel-independent pathways, and they can regulate directed cell migration, diapedesis, and ROS dissipation. In contrast to Cx30 which has a suppressive effect on the growth of malignant cells, Cx43 facilitates the migration and selective expansion of invasive glioma cells [38,39,40]. For instance, Cx43 is involved in the regulation of invadopodia in glioblastoma cells [41]. Direct cell-to-cell communication via Cx43 gap junctions can also induce glioma invasive behavior via gap junction-mediated microRNA signaling with astrocytes [42,43]. Recent findings have also demonstrated that Cx43 plays an important role in the microenvironment of malignant glioma [44], and, show that it is capable of conferring chemotherapeutic resistance to GBM cells [45,46]. Interestingly, temozolomide treatment significantly induced EMT markers in GBM cells [33]. On the other hand, the interrelations between EMT/GMT and Cx43 have not yet been analyzed in relation to the chemotherapy-induced microevolution of GBM.

The activation of self-defense mechanisms in tumor cells, such as cell dormancy, drug-efflux/metabolic, and DNA repair systems, reduces the sensitivity of GBM cells to chemotherapeutics. Among them, temozolomide (TMZ) acts as an alkylating factor that is capable of penetrating blood-brain barrier, which exerts a cytostatic effect via the methylation of guanine and the induction of base-pair mismatches during DNA replication. TMZ can also modify its pattern in GBM cells due to the involvement of DNA/histone methylation in the regulation of gene expression. The efficiency of de-methylation systems is crucial for the welfare of TMZ-treated cells. In particular, the activation of de-alkylation systems, such as O6-Methylguanine-DNA Methyltransferase (MGMT), counteracts TMZ effects and facilitates the expression of cell survival-promoting genes. The survival rates of TMZ-treated GBM patients correlate with the hypermethylation of MGMT promoter region in GBM tumor cells [47,48]. However, the involvement of MGMT-dependent chemoresistance in TMZ-induced GBM microevolution and its links with GMT/Cx43-related acquisition of invasive potential remain obscure. Here, we traced TMZ-induced pro-invasive phenotypic microevolution of GBM cells and its links with MGMT/Snail-1/Cx43-dependent adaptation to chemotherapeutic stress. In particular, we focused on (i) the phenotypic shifts of GBM cells under the long-term TMZ stress. Furthermore, we correlated them with (ii) the Cx43 levels and (iii) the activity of MGMT-dependent drug-resistance systems. Our data show that MGMT activity can provide the background for TMZ-induced Snail-1/Cx43-depedent microevolution of GBM invasive front.

## 2. Results

### 2.1. TMZ Increases the Invasive Potential of GBM Cells in a MGMT^−^ Independent Manner

**Figure 1:**
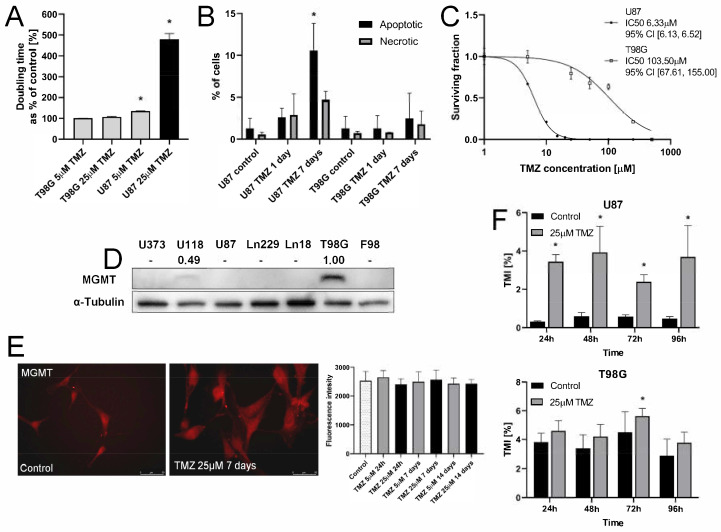
Sensitivity of T98G and U87 cells to temozolomide (TMZ). (**A**) U87 and T98G cells were incubated in the presence of 5 or 25 µM TMZ. Their proliferation was estimated after the next 24–96 h with Coulter Counter to calculate the population doubling times (for growth curves cf. Figure S1A). (**B**) Cells were treated with 25 µM TMZ and their apoptotic response was estimated after 24 h and seven days with AnnexinV/PI assay. At least 5000 single cells were analyzed, gated according to their area/aspect ratio (for original dot-plots, see Figure S1B). (**C**) Dose-dependence of cell survival under TMZ stress estimated with clonogenic assay. (**D**) O6-Methylguanine-DNA Methyltransferase (MGMT) levels in T98G and U87 cells cultivated in control conditions were estimated with immunoblotting. Numerical data show the density of MGMT-specific bands intensity calculated against the levels of α-tubulin as the housekeeping gene. (**E**) Cytofluorimetric analyses of TMZ effect on MGMT levels in U87 cells undergone 25 μM TMZ treatment (see also Figure S1C). Scale bar 50 μm (**F**) The effect of TMZ on the efficiency of T98G/U87 transmigration through microporous membranes (Transwell assay). Statistical significance was calculated with ANOVA/non-parametric Mann–Whitney test, * *p* < 0.05 vs. control. Data representative for three independent experiments. Note the correlation between MGMT levels and TMZ-resistance of U87 and T98G cells.

U87 and T98G cells display considerable differences in the reactivity to TMZ. In contrast to T98G cells, their U87 counterparts were highly reactive to TMZ. This is illustrated by a prominent inhibition of U87 proliferation (Figure S1A), the prolongation of U87 doubling-time (Figure 1A), and distinct TMZ-induced apoptotic response (Figure 1B; cf. Figure S1B). These effects were observed within seven days of incubation in the presence of TMZ administered at the concentrations of 5 and 25 μM, i.e., within the range of its therapeutically relevant doses. Consequently, the survival rate of U87 cells under TMZ stress was considerably lower than that of T98G cells, as shown by IC_50_ values that were estimated with the clonogenic assay (103.5 μM (T98G) vs. 6.33 μM (U87); Figure 1C). Relatively high TMZ-resistance of T98G cells (Figure 1A,C) was correlated with the relatively sparse fractions of apoptotic cells in TMZ-treated T98G populations (Figure 1B). The differences in TMZ-resistance between T98G and U87 cells can be ascribed to the MGMT-negative phenotype of U87 cells (Figure 1D). Actually, T98G expressed high MGMT levels, whereas we did not observe any significant increase of the MGMT levels in U87 cells after the TMZ treatment (Figure 1E). Despite these differences, an induction of U87 and T98G invasiveness was observed after TMZ application (Figure 1F). These data indicate that TMZ induces the microevolution of GBM invasiveness in a manner independent of MGMT.

### 2.2. TMZ Transiently Enhances Invasiveness of MGMT^low^ Cells

**Figure 2:**
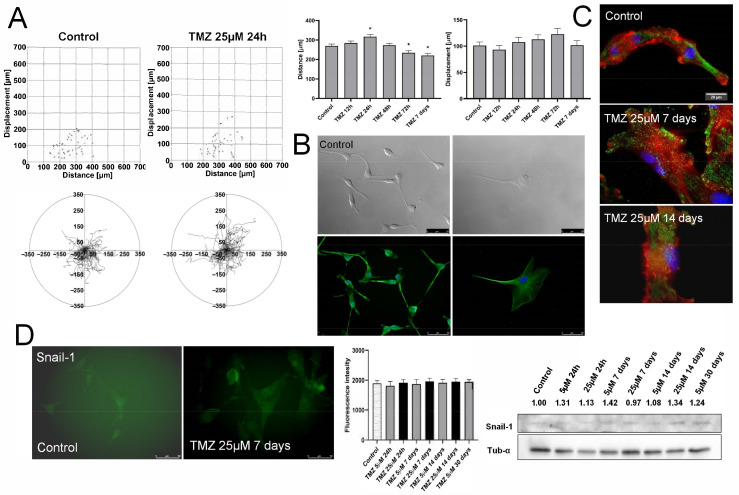
Effect of TMZ stress on the invasiveness of U87 cells. (**A**) U87 cells were seeded at the density of 2000/well of 12-well plate and incubated for 24 h before the administration of TMZ. Their motility was estimated with time-lapse video microscopy at the indicated time-points. Circular diagrams, dot-plots (for the data from the long-term TMZ treatment, see Figure S2A) and bar graphs show trajectories, movement parameters (distance and displacement) at the single cell and population level, respectively. (**B**) Cells were incubated in the presence of 25 μM TMZ, fixed, permeabilized and stained against α-tubulin (green)/DNA (blue) to visualize cell morphology/microtubular architecture at the indicated time-points. (cf. Figure S2B). Scale bar 50 μm. (**C**) U87 cells were incubated in the presence of 25 μM TMZ for 7/14 days and stained against vinculin (green)/actin (red)/DNA (blue). Scale bar 20 μm. (**D**) Effect of TMZ on the expression levels of Snail-1 in U87 populations. Snail-1 levels were then estimated with microscopy-assisted fluorimetry (left panel) and immunoblotting (right panel, cf. Figure S2C). Scale bar 50 μm. Numerical data show the density of Snail-1-specific bands intensity calculated against the levels of α-tubulin as the housekeeping gene. Statistical significance was calculated with ANOVA/non-parametric Mann–Whitney test, * *p* < 0.05 vs. control. Data representative for three independent experiments. Note that TMZ stress only transiently increases the invasiveness of U87 cells.

We focused on the potentially pro-invasive phenotypic shifts in TMZ-treated U87 populations to further identify the consequences of TMZ stress for the phenotype of GBM cells. The short-term administration of 25 μM TMZ increased the motility and displacement efficiency of U87 cells, followed by the gradual reversal of this effect starting after 48–72 h (Figure 2A). An enriched sub-population of highly motile U87 cells seen after 72 h of TMZ treatment remains in concordance with the data from transmigration assays, which revealed increased the fraction of invasive U87 cells (Figure 1E) that apparently constitutes the U87 “invasive front”. These effects were followed by the shifts of TMZ-treated U87 cells towards an “epithelioid” morphology (Figure 2B). Namely, spindle-like U87 cells were replaced by increasing the fraction of well spread, hypertrophic cells, reminiscent of the dormant “giant” cells that were observed elsewhere [49].

A corresponding, although less pronounced, effect was observed in the presence of 5 μM TMZ (Figure S2). Concomitantly, gradual phenotypic shifts of TMZ-treated U87 cells were accompanied by the rearrangements of their cytoskeleton architecture, incl. the maturation of vinculin^+^ focal adhesions (Figure 2C). Even though we could not observe any changes of Snail-1 levels in TMZ-treated U87 cells (Figure 2D), our data show a transient TMZ-triggered expansion of invasive U87 lineages. It is apparently attenuated by the cytotoxic TMZ effects in MGMT^low^ cells (cf. Figure 1A,B). Thus, the MGMT function is necessary for TMZ-induced microevolution of functional GBM lineages.

### 2.3. MGMT Facilitates Snail-1 Dependent Microevolution of GBM Invasiveness

**Figure 3:**
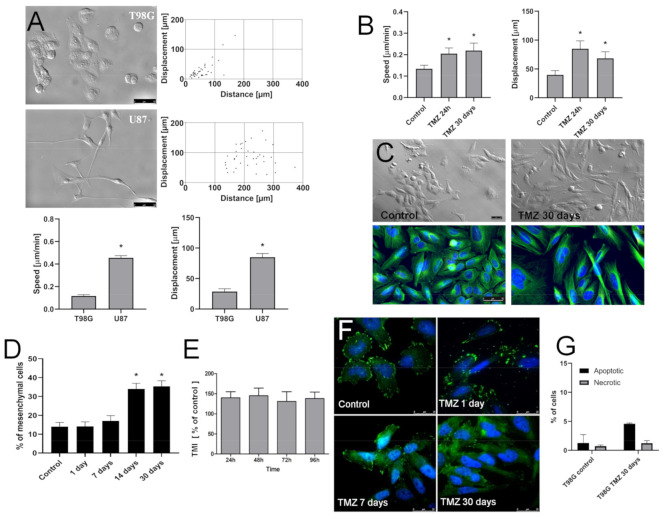
Long-term microevolution of invasive T98G cells under TMZ stress. (**A**) Comparison of innate morphology and motility of U87 and T98G cells estimated with immunofluorescence microscopy (tubulin-green/DNA-blue), Nomarski Interference Contrast (NIC) and time-lapse videomicroscopy. Dot-plots and bar graphs show trajectories, movement parameters at the single cell and population level, respectively (for circular diagrams, see Figure S3A). Scale bar 50 μm. (**B**,**C**) T98G cells were cultivated in the presence of 25 μM TMZ for one to 30 days. Their motility (**B**) and morphology (**C**) was estimated with time-lapse video and (NIC) microscopy, respectively. Architecture of microtubular cytoskeleton (tubulin-green/DNA-blue) was estimated with immunofluorescence (see also Figure S3B,C). Scale bar 50 μm. (**D**) T98G cells were cultivated in the presence of 25 μM TMZ for 1–30 days. The fractions of mesenchymal (rear-front polarized) cells (upper panel) and Snail-1 levels (lower panel) were then estimated with morphometric and immunofluorescence assays. (**E**) Invasive potential of T98G cells undergone the long-term (30 days) TMZ-treatment in vitro. Cells were seeded into the inserts, the number of cells that transmigrated through the membranes was estimated at the indicated time points and calculated in relation to naive T98G cells. (**F**) Naive T98G cells and their TMZ-treated (one, seven, and 30 days) counterparts were prepared as in Figure 1A and Cx43 levels were analyzed with immunofluorescence. Scale bar 25 μm. (**G**) Cells were treated with 25 µM TMZ for one and 30 days and their apoptotic response was estimated with AnnexinV/PI assay. At least 5000 single cells were analyzed, gated according to their area/aspect ratio. Statistical significance was calculated with ANOVA/non-parametric Mann–Whitney test, * *p* < 0.05 vs. control. Data are representative of three independent experiments. Note the relatively TMZ-resistance of MGMT^high^ T98G cells and the microevolution of invasive Cx43^high^ T98G lineages under persistent TMZ stress.

Further studies were performed to estimate the role of “innate” MGMT-dependent drug-resistance in the long-term microevolution of GBM cell invasiveness under TMZ stress. A comparison of U87 and T98G morphology/motility revealed a lower motility of MGMT^high^ T98G cells (Figure 3A).

In contrast to homogeneous, predominantly spindle-shaped MGMT^low^ U87 cells, a co-existence of epithelioid and fibroblastoid/mesenchymal cells was observed in MGMT^high^ T98G populations under the control conditions. This makes them a suitable model for the microevolutionary studies. The heterogeneous phenotype of T98G cells was accompanied by a sub-population of relatively fast-migrating T98G cells. Similarly to their U87 counterparts (cf. Figure 2), T98G cells reacted to the short-term TMZ treatment with an acceleration of their motility (Figure 3B). The prolongation of TMZ treatment did not result in T98G cell hypertrophy/flattening/senescence (as in the case of U87 cells). Instead, phenotypic shifts towards “fibroblastoid” morphology were detected by NIC and immunofluorescence microscopy (Figure 3C). This is summarized by morphometric analyses, which revealed an increased abundance of rear-front polarized cells in the long-term (30 days) TMZ-treated T98G populations (Figure 3D). Concomitantly, we observed an increased invasiveness and a considerable Cx43 up-regulation in these cells, as manifested by transmigration (Figure 3E) and immunoassays, respectively (Figure 3F). These pro-invasive effects of the long-term TMZ treatment were accompanied by a relatively low fraction of apoptotic cells in the populations that were cultivated in the presence of 25 μM TMZ (Figure 3G). Thus, TMZ induces the microevolution of invasive subsets of Snail-1^+^/Cx43^+^/MGMT^high^ T98G cells.

### 2.4. Snail-1 Is Crucial for TMZ-Induced Microevolution of the Invasive Subsets of T98G Cells 

**Figure 4:**
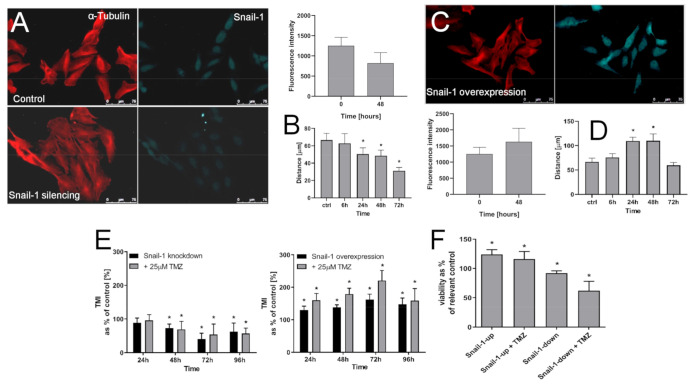
Effect of Snail-1 down/up-regulation on T98G phenotype. (**A**) T98G cells were transfected with Snail-1 targeting siRNA, followed by cytofluorimetric (FACS) estimation of intracellular Snail-1 levels and the analyses of cell morphology (right). Scale bar 75 μm. (**B**) Time-lapse estimation of T98G motility estimated immediately 6, 24, 72 and 96 h after transfection. (**C**,**D**) T98G cells were transduced with Snai-1 expression vector. Their Snail-1 levels, morphology (**C**), and motility (**D**) was estimated as in (**A**,**B**). Scale bar 75 μm. (**E**,**F**) The effect of 25 μM TMZ on the transmigration efficiency (TransMigration Index; (**E**)) and viability (**F**) of T98G cells undergone Snail-1 down/up-regulation. Statistical significance was calculated with ANOVA/non-parametric Mann–Whitney test, * *p* < 0.05 vs. control. Data are representative for three independent experiments. Note the synergy of Snail-1/TMZ effects on T98G invasiveness.

Concomitant induction of the invasiveness and Cx43 levels in MGMT^high^ T98G cells that were subjected to the long-term TMZ stress may suggest the involvement of Snail-1-dependent signaling in the microevolution of GBM invasive front. Therefore, we looked into the involvement of Snail-1 in TMZ-induced pro-invasive phenotypic shifts of T98G cells. After a transient siRNA-induced down-regulation of Snail-1 in these cells, its levels reached ca. 60% of the control value (Figure 4A). It was sufficient to evoke the morphological transition of T98G cells towards “epithelioid” phenotype and significantly attenuate their motility (Figure 4B).

Snail-1 up-regulation experiments further confirmed the notion on the crucial role of Snail-1 in the regulation of T98G invasiveness. Ectopically increased Snail-1 levels in T98G cells correlated with their shifts towards fibroblastoid morphology (Figure 4C) and with their considerably enhanced motility (Figure 4D). Furthermore, Transwell assays revealed the correlation between Snail-1 levels and the transmigration index (TMI) of T98G cells. This is illustrated by a gradual decrease of this parameter, along with Snail-1 down-regulation and the opposite effect after Snail-1 up-regulation (Figure 4E). Notably, we did not observe any effect of TMZ on the transmigration efficiency of T98G cells after Snail-1 silencing, whereas TMZ considerably augmented this parameter after Snail-1 up-regulation. These effects were accompanied by the correlation between Snail-1 levels and TMZ-resistance of T98G cells, as illustrated by the reduced viability of TMZ-treated T98G cells undergoing Snail-1 knock-down (Figure 4F). Collectively, our data confirm that Snail-1-dependent signaling participates in TMZ-induced invasiveness of T98G cells.

### 2.5. T98G Cells Display Sensitivity to Cx43 Down-Regulation

**Figure 5:**
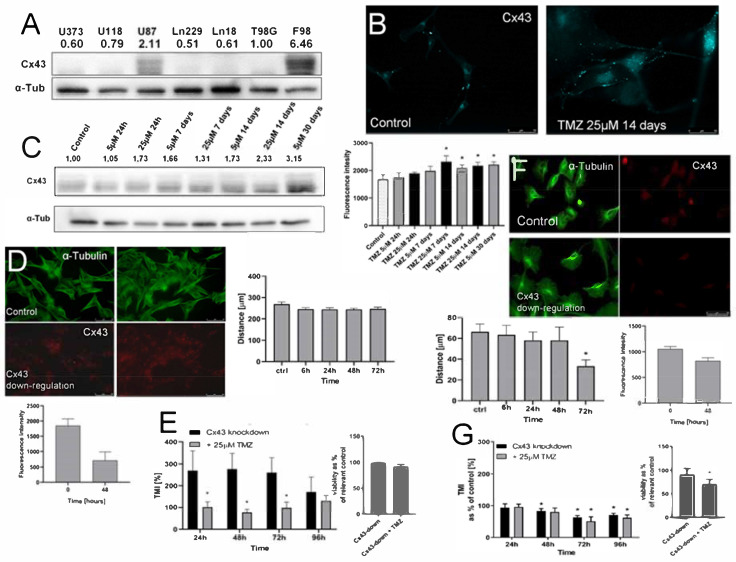
The effect of Cx43 down-regulation on the invasiveness of MGMT^high^ and MGMT^low^ cells. (**A**) Comparison of Cx43 levels in GBM cells estimated with immunoblotting. Numerical data show the density of Cx43-specific bands intensity calculated against the levels of α-tubulin as the housekeeping gene. (**B**,**C**) Effect of TMZ on the expression levels of Cx43 in U87 populations. Cells were treated with 5/25 μM TMZ for up to 30 days and Cx43 levels were estimated with fluorimetry ((**B**); see also Figure S4) and immunoblotting (**C**). Scale bar 50 μm. (**D**,**E**) Effect of Cx43 silencing in U87 cells on their motility (**D**), transmigration through microporous membranes and their viability (**E**). Scale bar 50 μm. (**F**,**G**) Effect of Cx43 silencing in T98G cells on their motility (**F**), and transmigration through microporous membranes and their viability (**G**). Scale bar 50 μm. Statistical significance was calculated with ANOVA/non-parametric Mann–Whitney test, * *p* < 0.05 vs. control. Data are representative for three independent experiments. Note the sensitivity of T98G cells to ectopic Cx43 down-regulation.

T98G and U87 cells constitute a suitable experimental tool for the analyses of the interrelations between MGMT and Snail-1 during TMZ-induced phenotypic GBM microevolution due to the differences in MGMT levels. Additionally, native U87 cells showed considerably higher levels of Cx43 than their T98G counterparts (Figure 5A). The application of TMZ considerably up-regulated Cx43 in U87 cells, as illustrated by cytofluorimetric analyses (Figure 5B) and immunoblotting (Figure 5C). However, an ectopic down-regulation of Cx43 had no effect on the motility of U87 cells (Figure 5D). Concomitantly, it reduced their transmigration potential in the presence of TMZ (Figure 5E), even though we observed an increase of otherwise very low U87 transmigration in its absence (cf. Figure 1F). In turn, morphological shifts of T98G cells towards epithelioid morphology and the inhibition of their motility was observed upon Cx43 down-regulation in T98G cells (Figure 5F). This effect corresponded to that observed after Snail-1 down-regulation (cf. Figure 4), and it was accompanied by increased sensitivity of T98G cells to TMZ (Figure 5G). It is illustrated by the attenuation of their invasiveness and viability after concomitant Cx43 down-regulation/TMZ-treatment. These data unequivocally show the involvement of Cx43 in the regulation of T98G invasiveness at the onset of its microevolution.

**Figure 6:**
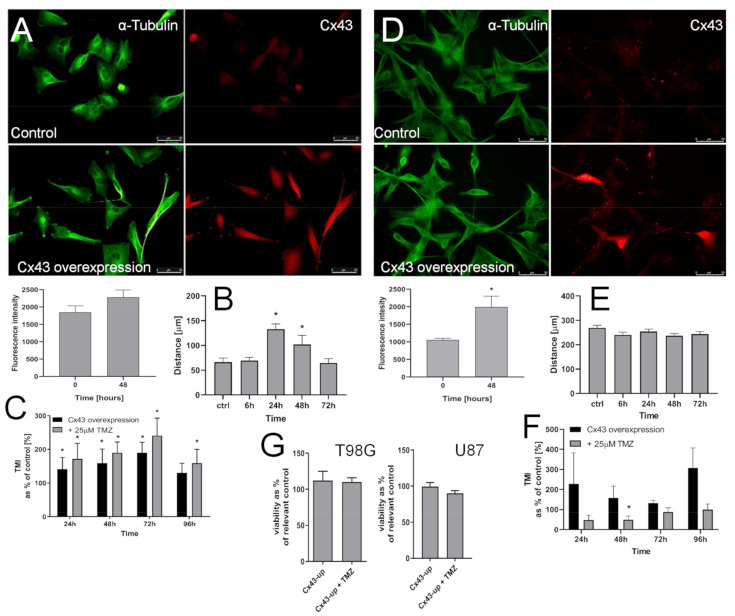
Effect of Cx43 up-regulation on the invasiveness of GBM cells. (**A**) T98G cells were transfected with Cx43 expression plasmid, followed by cytofluorimetric (FACS) estimation of intracellular Cx43 levels and the analyses of T98G morphology. Scale bar 50 μm. (**B**) T98G cells were processed as in A and their motility was estimated after 24, 48, and 72 h with time-lapse video microscopy. (**C**) The effect of Cx43 up-regulation on the transmigration of T98G cells through the microporous membranes in the presence of 25 μM TMZ. (**D**) Effect of Cx43 expression plasmid on the levels of Cx43 in U87 populations estimated with flow-cytometry. Scale bar 50 μm. (**E**,**F**) Time-lapse and Transwell estimation of U87 motility/invasiveness estimated six, 24, 48, 72, and 96 h after the onset of transduction with Cx43 expression vector. Scale bars 50 μm Statistical significance was calculated with non-parametric Mann-Whitney test, * *p* < 0.05 vs. control. Data representative for three independent experiments. Note the correlation between epithelial-mesenchymal transition (EMT)-related morphological changes, Cx43 up-regulation, and invasiveness of T98G cells in the presence of TMZ.

The effects of Cx43 up-regulation on the phenotype of T98G cells and their responsiveness to TMZ further confirmed this notion. Namely, transient Cx43 up-regulation in T98G cells (Figure 6A) increased their motility (Figure 6B). Concomitantly, TMZ exerted an enhancing effect on T98G transmigration potential (Figure 6C) after Cx43 up-regulation. These data remained in contrast to the negligible reactivity of MGMT^low^ U87 cells to the Cx43 signaling. This is illustrated by the lack of the effects of Cx43 up-regulation on their motility (Figure 6D,E) and their low invasiveness after concomitant Cx43 up-regulation and TMZ treatment (Figure 6F), even though an increase of otherwise very low U87 transmigration could be observed in its absence (cf. Figure 1F). Finally, we did not see any effects of Cx43 up-regulation on the viability of T98G and U87 cells (Figure 6G). Collectively, these observations show the MGMT-dependent involvement of Cx43 in the regulation of GBM invasiveness under TMZ stress.

## 3. Discussion

The prolonged administration of chemotherapeutic drugs in GBM treatment induces systemic adverse effects that (i) enforce premature cessations of tumor treatment and (ii) promote the “survival of the fittest” cells [50]. It potentially leads to the selective intratumoral microevolution/expansion of drug-resistant tumor cell lineages. Numerous data have identified the interrelations between the invasiveness and drug-resistance of tumor cells. These interrelations can facilitate the expansion of invasive GBM cell lineages. Consequently, a gradual increase of chemotherapeutic doses is enforced against continuously more aggressive tumor cells that reside within the weakening organism of the patient. Our current data indicate that Cx43 signaling enhances the invasiveness of GBM cells under TMZ stress in both MGMT^high^/MGMT^low^ populations. However, MGMT is needed to overcome TMZ toxicity, which allows for the establishment of phenotypically stable/expanding, drug-resistant, and invasive GBM cell lineages. Thus, we provide a novel scenario that shows MGMT importance for the selective expansion of invasive GBM cell lineages. Our data also illustrate the possible role of Snail-1/Cx43-dependent signaling in this process and GBM progression.

The long-term monitoring of the cell phenotype after chemotherapeutic stress gives information on cellular microevolution in vitro. We have previously used such an approach to elucidate Cx43 functions in the microevolution of prostate cancer cells [37] and estimate the involvement of cancer stem cells in DCX-induced microevolution of prostate cancer drug-resistance [51]. Here, we combined short- and long-term analyses of GBM cell behavior to demonstrate the increased invasiveness of GBM cells that are capable of surviving the short-term TMZ stress. This effect was also observed after the long-term TMZ treatment of T98G, but not U87 cells in vitro and in vivo (data not shown). Conceivably, it illustrates the phenomena that underlie the microevolution of GBM malignancy under TMZ stress. TMZ-induced phenotypic microevolution of GBM cells can be ascribed to (i) the selective survival/expansion of pre-existing TMZ-resistant T98G cells and/or (ii) the induction of pro-invasive phenotypic shifts under TMZ stress. Notwithstanding the mechanism, an increased fraction of rear-front polarized cells indicates that glial-mesenchymal transition (GMT) may participate in the potentially pro-invasive activity of TMZ. Corresponding morphologic shifts, an augmented invasiveness of T98G cells upon the ectopic over-expression of Snail-1 in the presence of TMZ, and the opposite cell reactions after Snail-1 down-regulation confirm this notion. They remain in agreement with previous reports on the role of TMZ, Snail-1, and EMT inducers in the regulation of GBM invasiveness [52,53,54,55,56]. However, our study is probably the first to discuss the function of Snail-1 in terms of TMZ stress-induced microevolution of GBM invasiveness. These in vitro data need to be verified in vivo; however, at the current stage of the research, they illustrate the mechanistic link between Snail-1 signaling and chemotherapy-induced microevolution of GBM invasiveness.

Numerous co-factors participate in Snail-1-dependent EMT/GMT of tumor cells. For instance, Cx43 was shown to regulate TGF/Snail-1-induced EMT in prostate cancer cells [37]. Channel-dependent and channel-independent functions of Cx43 have both been described in tumor promotion and progression [57]. In the central nervous system, Cx43 function has been implicated in the development of GBM. We present at least four lines of evidence that confirm the links between Cx43 expression and TMZ-induced microevolution of GBM invasiveness. First, Cx43 up-regulation was observed in TMZ-treated T98G and U87 cells. Together with the impairment of T98G invasive phenotype after Cx43 down-regulation and with the opposite effect of ectopic Cx43 up-regulation, it identifies Cx43 as the linker between Snail-1-dependent reactivity of GBM cells to TMZ and their invasiveness. Enhanced T98G motility under TMZ stress after Cx43 up-regulation further confirmed this notion. Finally, the interrelations between the reactivity of T98G cells to Snail-1/Cx43 manipulations suggest the importance of the positive feedback loop between Cx43 and Snail-1 signaling for the microevolution of T98G cells. A corresponding loop has been observed in prostate cancer cells, where Cx43 was shown to regulate the activity of TGF-induced signaling pathways via the interference with the sequestration of Smad2/3 on microtubules [37,58]. The involvement of Cx43 and Cx43-mediated intercellular communication during the chemotherapy-induced microevolution of GBM chemoresistance remains to be precisely elucidated. However, our current data reveal a novel function of Cx43 in the progression of GBM. Cx43 can participate in the formation of GBM invasive front partly in a Snail-1-dependent manner, being a candidate for a predictive factor in GBM diagnostics. 

On the other hand, Cx43^high^/MGMT^low^ U87 cells were less predestined to microevolve under TMZ stress, which indicates that Cx43 is not sufficient for this process. Cx43 up-regulation was still observed in TMZ-treated cells. Therefore, it can act to enhance *en masse* U87 drug-resistance in a GJIC-dependent manner via promoting their metabolic cooperation and detoxification (due to the intercellular dissipation of ROS and exchange of ROS scavengers [40]). However, an enhancement of otherwise very low U87 transmigration efficiency was observed both after the ectopic Cx43 down- and up-regulation. Presumably, Cx43 manipulation prompts the transmigration of discrete U87 lineages, which are differentially sensitive to Cx43 signaling. Whereas, this interesting finding requires further study, differences in the reactivity of MGMT^high^ T98G, and MGMT^low^ U87 cells to TMZ, in particular the inhibition of U87 invasiveness after Cx43 up-regulation (as compared to the opposite effect in T98G populations), suggests that MGMT is a prerequisite for Cx43-dependent/TMZ-induced microevolution of invasive GBM lineages. MGMT removes alkyl groups from O6 position in guanine, thus counteracting the pro-apoptotic and cytotoxic activity of TMZ [30,31]. A correlation between the innate drug-resistance and Cx43-dependent reactivity of GBM cells to TMZ is also illustrated by the signs of dormancy/senescence, being accompanied by the hypertrophy and epithelioid morphology after the long-term TMZ-treatment of U87 cells. Whether a dysfunction of the feedback loop between Cx43 and Snail-1 in U87 cells participates in this effect requires further study. In any case, our data show the existence of a novel MGMT/(Snail-1)/Cx43-dependent system that gives way to the expansion of invasive GBM sub-populations in vitro and in vivo.

The balance between “secular” phenotypic transitions and permanent reprogramming of tumor cells underlies the processes of their phenotypic diversification and tumor microevolution. Collectively, our data revealed a Cx43-dependent mechanism of GBM adaptation to the chemotherapeutic stress and its mechanistic links with the microevolution of GBM invasiveness. It potentially involves integrated Snail-1/Cx43 signaling that can underlie the selective survival of GBM cells under TMZ stress and the subsequent formation of a GBM invasive front. MGMT apparently provides the background for the establishment of a Snail-1/Cx43-related positive feedback loop and it facilitates the establishment of phenotypically stable/expanding and invasive GBM lineages. These data illustrate the significance of MGMT/Snail-1/Cx43 interrelations for the invasive GBM front formation; however, they still leave several open questions. Further studies are necessary to (i) identify the mechanisms of TMZ-induced Cx43/Snail-1 up-regulation in GBM cells, (ii) to scrutinize the interrelations between MGMT and Cx43-dependent GBM drug-resistance, and (iii) to elucidate the contribution of single cell adaptation to TMZ versus selective elimination of drug-sensitive cells by TMZ during the microevolution of GBM lineages. Most importantly, the mechanisms underlying differential MGMT up-regulation in discrete GBM lineages and their links to the functional status of Cx43/Snail-1 require further studies. In any case, we provide new data on the significance of Cx43^+^ phenotype for GBM invasion and suggest a novel Cx43-dependent mechanism of GBM microevolution under the chemotherapeutic stress.

## 4. Materials and Methods

### 4.1. Cell Culture

Human GBM T98G (ATCC, CRL-1690) and U87 cells (ATCC, CRL-1690) were routinely cultivated in high glucose (4500 g/L) DMEM medium (Sigma, St. Louis, MO, USA), which was supplemented with 10% fetal bovine serum (FBS; Gibco) and 1% Antibiotic-Antimycotic Solution (Sigma) [59,60]. For endpoint experiments, the cells were harvested with 0.25% trypsin in Ca^2+^/Mg^2+^-free PBS (Corning, Corning, NY, USA) supplemented with 0.5 mM EDTA (UltraPure^TM^; Invitrogen, Carlsbad, CA, USA), counted with Z2 particle counter (Beckman-Coulter, Brea, CA, USA) and then seeded into multi-well tissue culture plates (Falcon). For some experiments U373 (human astrocytoma; ATCC, HTB-17), U118 (ATCC, HTB-15), Ln229 (ATCC, CRL-2611), Ln18 (ATCC, CRL-2610) and F98 cells (rat glioblastoma; ATCC, CRL-2397) were cultivated, as described above. TMZ (Sigma; No. T2577) was dissolved in DMSO at the concentration of 100 mM, stored in −80 °C, and administered at the concentrations of 5 or 25 μM. Unless stated otherwise, the medium containing 0.25‰ DMSO (corresponding to 25 μM TMZ) was used as a vesicle control.

### 4.2. Cell Proliferation, Apoptosis and Viability

T98G and U87 cells were seeded into 12-well plates (Falcon) at the density of 5.3 × 10^4^ cells/cm^2^ and allowed to attach, before the administration of 5 or 25 µM TMZ-supplemented medium. Subsequently, the cells were harvested every 24 h for the next four days (96 h) and counted in a Z2 particle counter. The obtained data were processed with GraphPad Prism software to calculate the kinetics of cell proliferation (incl. the doubling times). For the analyses of apoptosis, the cells were cultured in T-25 flasks (Falcon) in the presence of 25 µM TMZ for up to 30 days, trypsinized, re-suspended in Ca^2+^/Mg^2+^-free PBS, and subjected to AnnexinV/propidium iodide staining according to the manufacturer’s protocol (BD Pharmigen, Franklin Lakes, NJ, USA). Flow cytometric detection of apoptotic cells was performed with ImageStreamX imaging cytometer (Merck, Darmstadt, Germany). For each condition, at least 5000 singlets were collected. The data were processed using IDEAS 6.2 software (Merck; [59]). The biability of cells was analyzed using Trypan blue assay (Sigma; No. T8154) assay, as described before [37]. For the clonogenic assay, the cells were seeded into six-well plates (500 cells/well) and then incubated in the presence of 0–500 µM TMZ (or 0.5% DMSO) to form colonies for two weeks. Subsequently, the samples were fixated with formaldehyde (FA; 3.7%; 20 min at 37 °C), washed, stained with 0.01% Crystal Violet solution in EtOH, dried, and the colonies were counted. The data were processed with GrapPad Prism software to calculate IC50.

### 4.3. Cell Motility and Transmigration

T98G/U87 cell movement was recorded with the Leica DMI6000B time-lapse video microscopy system, equipped with an incubation chamber (37 °C ± 0.2 °C)/(5% CO_2_), IMC contrast optics, and DFC360FX CCD camera. The cells were seeded into 12-well plates at the density of 500 cells/cm², immersed in TMZ-containing medium for 48 h and their movements were recorded for the next eight hours (time step—10 min). The cell trajectories were constructed from a sequence of cell centroid positions recorded for eight hours at 300 s time intervals (using a dry ×20, NA—0.4 objective) to estimate the length of single cell trajectory (Distance; μm) and the single cell displacement (Displacement; μm). Cell trajectories were pooled to calculate the averaged values of Distance and Displacement at the population level (at least three independent experiments; *n* = 50 [61]). Invasive potential of T98G and U87 cells was examined with transmigration assay using the Transwell^TM^ inserts (8 µm micropores; Corning). The cells were seeded onto the top of microporous membrane at the density of 1 × 10^4^ cells/24-well plate compatible insert and allowed to transmigrate for 24–96 h in the absence/presence of TMZ, allowed to grow for the next 72 h, and then harvested and counted with Z2 particle counter. Transmigration index (TMI) was calculated as the percentage of transmigrating cells/population normalized against the time of transmigration (24–96 h) and cell proliferation rates [51].

### 4.4. Immunofluorescence

Immunolocalization of Cx43, Snail-1, MGMT, vinculin, and α-tubulin was performed in formaldehyde/Triton X-100 fixed/permeabilized cells (FA; 3.7%; 20 min at 37 °C)/Triton X-100 (0.1%; 10 min at RT). Non-specific binding sites were then blocked with 2% BSA (30–45 min, RT), followed by the incubation of specimens in the presence of primary antibodies: mouse anti-vinculin IgG (NV9131, Sigma, 1:300), mouse anti-α-tubulin IgG (Sigma, No. T9026, 1:500) rabbit anti-Cx43 IgG (Sigma; No. C6219; 1:500), and goat anti-Snail-1 *N*-terminal IgG (Sigma; No. SAB2501370; mouse anti-O6-Methylguanine-DNA Methyltransferase (Sigma). Subsequently, the cells were labeled with AlexaFluor488-conjugated donkey anti-mouse IgG (No. A21202, Invitrogen, Carlsbad, CA, USA) or AlexaFluor647-conjugated chicken anti-rabbit IgG (Invitrogen; No. A21443), AlexaFluor546-conjugated donkey anti-mouse IgG (Invitrogen; No. A10036), and AlexaFluor488-conjugated chicken anti-goat IgG (Invitrogen; No. A21467). In places, the cells were counterstained with Alexa546-conjugated phalloidin (Invitrogen; No. A22283) and Hoechst 33258 (No. B2883, Sigma) [62]. The images were acquired with Leica DMI6000B fluorescence microscope equipped with a DFC360FX CCD camera, total internal reflection fluorescence (TIRF) module (DMI7000 version; Leica Microsystems, Wetzlar, Germany), Nomarski interference contrast (DIC), and IMC modules using ×40, NA-1.47 or ×100 oil immersion objectives, and the Leica Application Suite Advanced Fluorescence software [37]. The raw images were additionally processed (contrast adjustment, background substraction, fluorimetric analysis) with ImageJ software. When indicated, cells were suspended with 0.2% EDTA in Ca/Mg-free PBS, fixed, permeabilized, and stained in suspension, as described above, and flow cytometric detection of Snail-1/Cx43 cells was performed with ImageStreamX imaging cytometer (Merck). For each condition, at least 5000 singlets were collected. The data were processed using IDEAS 6.2 software.

### 4.5. Transient Cx43/Snail-1 down/up-Regulation

T98G/U87 cells were were seeded at the density of 5000 cells/cm^2^ in a 12-well plate in antibiotic-free DMEM medium that was supplemented with 10% FBS and grown at 37 °C for 24 h. Subsequently, the medium was replaced with Opti-MEM Reduced Serum Medium (31985070, Gibco-Life Technologies, Waltham, MA, USA) and cells were treated with complexes of 1.5 μL Lipofectamine™ 2000 Reagent (11668-019, Invitrogen) and 1 μg plasmid DNA/small interfering RNA (siRNA) in Opti-MEM, according to the manufacturer’s protocol. Afterwards, the medium was replaced by standard DMEM medium with 10% fetal bovine serum and 1% Antibiotic-Antimycotic Solution and the efficiency of Cx43/Snail-1 up/down-regulation was analyzed with immunofluorescence-assisted cytofluorimetry. The following siRNA/plasmids were used: SNAI-1 siRNA and siRNA-A (sc-38398 and sc-37007, Santa Cruz Biotechnology, Dallas, TX, USA), Snail_pGL2 (#31694, Addgene, Watertown, MA, USA), Cx43_FLAG (#17664, Addgene), MISSIONR esiRNA GJA1 (Sigma-Aldrich, St. Louis, MO, USA), and Cx43 shRNA (sc-29276-SH, Santa Cruz Biotechnology). The cells were subjected to endpoint experiments within the next 96 h. Cx43/Snail-1 expression was monitored with flow cytometry (see Section 2.4).

### 4.6. Immunoblotting

For the analyses of intracellular Snail-1/Cx43/MGMT levels, the cells were incubated in the absence/presence of TMZ, as indicated in the text, harvested with cold (~4 °C) Ca^2+^/Mg^2+^—free PBS/EDTA solution, centrifuged, and dissolved in a Triton X-100 cell lysis buffer with protease inhibitor cocktail (followed by freeze-thaw procedure and sonication). Bradford assay was applied for the estimation of total protein content in the lysates. The samples (20 µg of protein) were separated on 12% polyacrylamide gel (SDS-PAGE electrophoresis; Laemmli protocol; [37]), followed by the electrotransfer of proteins into PVDF membranes (Immun-Blot^®^ PVDF Membrane, #1620177; Bio-Rad, Hercules, CA, USA). Membranes were then blocked with skimmed milk/TBST solution and then incubated in the primary antibodies: monoclonal goat anti-Snail-1 IgG (Sigma; SAB2501370; 1:500), polyclonal rabbit anti-Cx43 (Sigma; No. C6219; 1:3000) or monoclonal mouse anti-MGMT IgG (Sigma; MAB16200; 1:500). α-tubulin was labeled with monoclonal IgG mouse anti-α-tubulin antibody (Sigma; No. T9026; 1:1000) and it used as a reference protein. Signal detection was performed with HRP-conjugated goat anti-rabbit IgG (No. 31466, Thermo Fisher Scientific, Waltham, MA, USA) and goat anti-mouse IgG (Thermo Fisher Scientific; No. 31430) using the chemiluminescence technique (Merck, Luminata Crescendo; No. WBLUR0500). For membrane imaging, MicroChemii system (DNR Bio-Imaging System) was used.

### 4.7. Statistical Analysis

Statistical significance of differences was tested with non-parametric Mann–Whitney U-test. Analyses were performed using Origin 2020 software.

## 5. Conclusions

MGMT has long been known to counteract the alkylating activity of TMZ. Our current data indicate that MGMT activity can be a prerequisite for the selective expansion of invasive GBM lineages, even though MGMT-related GBM chemoresistance is not necessary for its initiation. Phenotypic shifts of GBM cells that were observed under the long-term TMZ stress and their dependence on Cx43/Snail-1 levels demonstrate that the activation of Snail-1/Cx43-depedent signaling is required for TMZ-induced microevolution of invasive GBM lineage(s). This potentially illustrates the significance of MGMT/Snail-1/Cx43 interrelations for the invasive GBM front formation. Momentarily, it is premature to conclude on the clinical consequences of these interrelations; therefore, our in vitro data need to be verified in vivo. However, we describe a novel scenario of GBM microevolution towards malignancy under chemotherapeutic stress.

## Data Availability

The data that supports the findings of this study are available in the supplementary material of this article or available from the corresponding author upon reasonable request.

## Supplementary Materials

The following are available online at https://www.mdpi.com/article/10.3390/ijms22084150/s1, Figure S1. Effect of TMZ on the proliferation, apoptosis and MGMT levels in T98G and U87 cells, Figure S2. Effect of TMZ on the motility, morphology/architecture of microtubular cytoskeleton and Snail-1 levels in U87 cells, Figure S3. Motility and morphology of T98G and U87 cells. Figure S4. Effect of TMZ on the expression levels of Cx43 in U87 populations.
